# Supplementary material for: Understanding the Experience of Canadian Women Living with Ovarian Cancer through the Every Woman StudyTM
Source: Curr Oncol. 2022 May 5;29(5):3318–40. doi: 10.3390/curroncol29050271 (PMC9139742; doi:10.3390/curroncol29050271)
Supplement: Supplementary file 1 [file curroncol-29-00271-s001.zip › curroncol-1680870-supplementary.pdf]

File S1. Survey questions and coding

Core questions = questions that all participants will see, regardless of answers
Contextual questions = questions that only appear depending on previous answers
Skip logic

About you

3. To determine the geographic region where you were initially diagnosed and treatment decisions were made, please provide the first three digits of the postal code of your home at the time you were initially diagnosed and treated. If you do not know the postal code, please include the name of your town and city where you lived at that time. ***[answer required]***

4. Are you First Nations, Métis or Inuk (Inuit)?

- No
- Yes, First Nations
- Yes, Métis
- Yes, Inuk (Inuit)

5. Please indicate your ethnicity. By ethnicity we mean your feeling of belonging and attachment to a distinct group of a larger population that shares their ancestry, colour, language or religion. Tick all that apply:

- Arab
- Black
- Chinese
- Filipino
- French Canadian
- Japanese
- Jewish
- Korean
- Latin American
- South Asian (e.g., East Indian, Pakistani, Sri Lankan)
- South East Asian (e.g., Vietnamese, Cambodian, Laotian, Thai)
- West Asian (e.g., Iranian, Afghan)
- Caucasian
- I'd rather not say
- Other, please specify:

6. What is your first language?

- English
- French
- Other, please specify:

7. In what year were you diagnosed with ovarian cancer? If you've been diagnosed with a recurrence, please put the year of your initial diagnosis.

8. What is the highest level of education you have completed?

- Elementary/Middle school
- High school
- Certificate program
- College/University/Post-graduate degree

9. When you were diagnosed with ovarian cancer, were you:

- Married or living with a partner
- Widowed
- Divorced or separated
- In a relationship but not living together
- Single (never married)
- Prefer not to say

10. When you were diagnosed, what was your total household income?

- <$25,000
- $25,000 - $49,999
- $50,000 - $74,999
- $75,000 – $99,999
- >$100,000
- Prefer not to say

Information on your ovarian cancer diagnosis

Please note that the term "ovarian cancer" in the following section refers to cancers diagnosed as ovarian, fallopian tube and/or primary peritoneal origin.

11. What age were you when you were first diagnosed with ovarian cancer?

12. What specific type of ovarian cancer were you diagnosed with?

- High grade serous
- Endometrioid
- Clear cell
- Mucinous
- Low grade serous
- Germ cell tumour
- Sex cord stromal tumour (ex: granulosa cell tumour)
- Borderline tumour
- My doctor never told me
- I do not know / I cannot remember
- Other (please specify):

13. What stage was your ovarian cancer at the time of diagnosis?

- Stage 1 (confined to the ovary/ovaries)

- Stage 2 (it had grown outside the ovary/ovaries, but only as far as the pelvis)
- Stage 3 (it had spread outside the pelvis into the abdominal cavity)
- Stage 4 (the cancer had spread into other body organs such as the liver or lungs)
- I do not know/I cannot remember

14. How did you find out this information on your type and/or stage of ovarian cancer?
- I asked for the information
- I was told without asking
- I read it in my pathology report
- I cannot remember
- Other

15. Are you currently:
- Undergoing treatment for newly diagnosed ovarian cancer
- Undergoing treatment for recurrent ovarian cancer
- Receiving maintenance or palliative therapy hoping to keep the cancer stable
- In remission (the cancer is not active)
- In palliative care to relieve symptoms
- I have chosen not to receive treatment/further treatment
- Other (please specify)

16. Since your initial diagnosis and treatment, has your ovarian cancer ever returned?
- No
- It has returned once
- It has returned twice
- It has returned three times or more
- My ovarian cancer never went away

Who pays for treatment

Within Canada, the drugs required for ovarian cancer treatment are not always covered by public health plans. The drugs that may or may not be covered can vary from province to province, as each province has its own individual health plan.

17. During your ovarian cancer treatments, were you required to personally assume/ pay for any of the costs for your treatments and/or drugs?
- Yes
- No ***[skip to Q19]***
- Not sure/don't remember

18. Was there ever a time when you were unable to receive treatment and/or drugs because you could not afford the cost?
- No ***[skip to Q20]***
- Not sure

- Yes, please specify

19. For any costs not covered by provincial and territorial public health plans, who has contributed towards the cost of your care and treatment? Tick all that apply:

- A private health insurance company
- My workplace (through an employee group insurance program)
- My workplace (directly)
- A pharmaceutical company's Patient Support (Compassionate) Program
- I have had to contribute directly
- Other family members and/or friends have contributed directly

20. Have you ever had to challenge what costs an insurance company will cover?

- Yes
- No ***[skip to Q22]***

21. Did making the application, or challenging the decision delay the start of treatment or change the course/type of treatment due to affordability?

- Yes
- No
- I don't know

Family history of cancer

22. Have any of the following family relatives (i.e. blood relatives) had cancer?
Tick all that apply:

- Ovarian
- Breast
- Uterine (Endometrial)
- Colorectal
- Prostate
- Pancreatic
- None of the listed cancers/ no cancer
- I don't know
- N/A

- Mother
- Father
- Daughter/s
- Son/s
- Sister/s
- Brother/s
- Aunt/s (your mother's or father's sibling)
- Uncle/s (your mother's or father's sibling)
- First Cousin/s (child of either mother's or father's sibling)
- Grandmother (mother's side)

- Grandfather (mother's side)
- Grandmother (father's side)
- Grandfather (father's side)
- Other more distant relatives (mother's side)
- Other more distant relatives (father's side)

Genetic testing

23. Have you ever been tested to see if you have a genetic mutation associated with an increased risk for ovarian or other cancers in families? (e.g. tests for the BRCA1 or BRCA2 gene)

- Yes, I was tested before I was diagnosed with ovarian cancer
- Yes, I was tested after I was diagnosed with ovarian cancer
- No, I have not been offered genetic testing ***[skip to Q26]***
- No, I do not wish to be tested ***[skip to Q26]***
- I'm not sure / I can't remember ***[skip to Q26]***

24. What were the results of your genetic test?

- A BRCA1 mutation was found
- A BRCA2 mutation was found
- No genetic mutations were found
- A variant of uncertain significance was found (inconclusive result)
- I'm not sure / I can't remember
- A mutation was found in a different gene

25. Did you have to pay to undergo genetic testing?

- No, it was covered by my provincial and territorial public health plan
- No, it was covered by an insurance company
- Yes, I or a relative or friend paid for the testing
- Other

Before you were diagnosed

26. Before you were diagnosed with ovarian cancer, how much if anything did you know about ovarian cancer?

- I knew a lot about it
- I knew a bit about it
- I had heard of it but didn't know anything about it
- I had never heard of it
- I don't remember

27. Thinking back to before your diagnosis, did you know that any of the following symptoms, if experienced frequently, could indicate someone might have ovarian cancer? Tick all those you knew could be symptoms of ovarian cancer:

- Bloating and/or increased abdominal size
- Abdominal pain or discomfort
- Urinary symptoms (increased frequency, increased sense of urgency)
- Difficulty eating (feeling full quickly)

- Changes in bowel habits (diarrhea and/or constipation)
- Menstrual irregularities
- Weight gain or loss without explanation
- Fatigue (extreme/persistent)
- None of the above

Leading up to your diagnosis

Investigation of symptoms

28. Leading up to your diagnosis with ovarian cancer, which if any, of the following symptoms did you experience? Tick all that apply:

- Persistent bloating and/or increased abdominal size ***[skip to Q30]***
- Abdominal pain or discomfort ***[skip to Q30]***
- Urinary symptoms (increased frequency, increased sense of urgency) ***[skip to Q30]***
- Difficulty eating (feeling full quickly) ***[skip to Q30]***
- Changes in bowel habits (diarrhea and/or constipation) ***[skip to Q30]***
- Menstrual irregularities ***[skip to Q30]***
- Weight gain or loss without explanation ***[skip to Q30]***
- Fatigue (extreme/persistent) ***[skip to Q30]***
- I did not experience any symptoms
- Other symptoms

29. As you did not experience symptoms, what led to your diagnosis? Tick all that apply:

- A routine examination ***[skip to Q43]***
- A routine scan ***[skip to Q43]***
- Ovarian cancer was discovered while I was being tested or treated for something else ***[skip to Q43]***
- Ovarian cancer was discovered during pregnancy or childbirth ***[skip to Q43]***
- Other ***[skip to Q43]***

30. How concerned were you about your symptoms before your diagnosis?

- Very concerned
- Fairly concerned
- Not very concerned
- Not at all concerned

31. Which symptom caused you the most concern?

- Persistent bloating and/or increased abdominal size
- Abdominal pain or discomfort
- Urinary symptoms (increased frequency, increased sense of urgency)
- Difficulty eating (feeling full quickly)
- Changes in bowel habits (diarrhea and/or constipation)
- Menstrual irregularities
- Weight gain or loss without explanation
- Fatigue (extreme/persistent)
- None in particular

- Other symptom

32. Did you consult a health professional about your symptoms?
- Yes
- No, it was something else that led to my diagnosis

33. Which type of health professional did you FIRST visit or talk to about your symptoms?
- A family doctor (GP)
- A doctor at an urgent care or drop-in clinic
- A doctor at an emergency department
- A gynecologist
- A gastroenterologist
- A nurse practitioner
- Other

34. How soon after experiencing symptoms did you first visit that health professional?
- Within one month of experiencing symptoms
- More than 1 month but less than 3 months
- More than 3 months, but less than 6 months
- More than 6 months, but less than 1 year
- More than 1 year after experiencing symptoms

35. In your view, how seriously did the health professional take your concerns when you first saw them?
- Very seriously
- Fairly seriously
- Not very seriously
- Not at all seriously
- I don't know / I cannot remember

36. When you first visited this health professional because of the symptoms you were experiencing, what did he or she think may be wrong with you? Tick all that apply:
- Cancer
- Irritable bowel syndrome
- A urinary infection
- Diverticulitis
- Menopausal problems
- Osteoporosis
- A hernia
- Ovarian cyst
- Gall stones
- Stress
- Food poisoning
- Gastric problems

- Depression
- Health issues related to weight
- Health professional did not say/thought it was nothing
- I do not know / I cannot remember
- Other

37. Did the health professional that you first went to regarding your symptoms order any tests to investigate your symptoms?
- Yes, right away ***[skip to Q39]***
- Yes, after further appointments with this first health professional
- No tests were ordered by the first health professional I saw and no referral to a specialist was made ***[skip to Q40]***
- No tests were ordered by the first health professional I saw but a referral to a specialist was made ***[skip to Q40]***

38. Since these tests weren't ordered right away, how long did it take from your first visit with this health professional to the tests being ordered (in months):

39. Which tests were ordered by the first health professional you went to? Tick all that apply:
- CA125 blood test
- Abdominal ultrasound (through your stomach wall)
- Trans-vaginal ultrasound (through a probe inserted into your vagina)
- Pelvic exam (where doctor inserts fingers in vagina and/or rectum to feel for abnormalities)
- MRI scan
- CT scan
- X-ray
- I do not know or cannot remember
- Other

40. Leading up to your diagnosis, what other types of health professionals did you see about your symptoms? Tick all that apply:
- A family doctor (GP)
- A gynecologist
- A gastroenterologist
- An emergency room doctor
- A nurse practitioner
- A doctor who specializes in treating ovarian cancer (gynecologic oncologist)
- I did not see anyone else
- I do not know / I cannot remember
- Another type of health professional, (please describe)

41. Did any of these other health professionals order any of the following tests?
Tick all that apply:
- CA125 blood test

- Abdominal ultrasound (through your stomach wall)
- Trans-vaginal ultrasound (through a probe inserted into your vagina)
- Pelvic exam (where doctor inserts fingers in vagina and/or rectum to feel for abnormalities)
- MRI scan
- CT scan
- X-ray
- I do not know or cannot remember
- Other

42. In your view, if you had important questions to ask health professionals about these tests that led to your diagnosis, did you get answers you understood?
- Yes, always
- Yes, sometimes
- No
- I had no need to ask questions
- I was not able to ask questions

Leading up to your diagnosis

Referral to a cancer specialist

43. Which health care professional referred you to a cancer specialist (e.g. gynecologic oncologist at a cancer centre):
- A family doctor (GP)
- A gynecologist
- A gastroenterologist
- An emergency room doctor
- A nurse practitioner
- I do not know / I cannot remember
- Another type of health professional, (please describe)

44. How much time passed in total from your FIRST visit to a health professional, to the time you were referred to a cancer specialist?
- Less than 1 month
- More than 1 month but less than 3 months
- More than 3 months but less than 6 months
- More than 6 months but less than 1 year
- More than 1 year but less than 2 years
- More than 2 years
- I don't know / I can't remember

45. How much time passed in total from your FIRST visit to a health professional, to the time you were given your diagnosis?
- Less than 1 month
- More than 1 month but less than 3 months
- More than 3 months but less than 6 months

- More than 6 months but less than 1 year
- More than 1 year but less than 2 years
- More than 2 years
- I don't know / I can't remember

Getting your diagnosis

46. Who told you of your initial diagnosis of ovarian cancer?

- An oncologist (cancer doctor)
- A gynaecologist
- An emergency room doctor
- A family doctor
- A nurse practitioner
- A member of your family
- Someone else

Information on diagnosis

47. When you were given your diagnosis, were you given ALL the information you needed at that time?

- Yes
- No, but they gave me some information
- No, they did not give me any information ***[skip to Q49]***
- I do not know / I cannot remember ***[skip to Q49]***
- I did not want or need any information ***[skip to Q50]***

48. What did the information cover? Tick all that apply:

- General information about ovarian cancer
- Treatments for ovarian cancer
- Information about living with cancer
- Genetic testing
- Sources of support for those with ovarian cancer (e.g. websites, telephone helpline, group meetings)
- Contact details for the health professionals involved in caring for you
- I do not know / I cannot remember
- Other

49. What would you have liked the information to cover? Tick all that apply:

- General information about ovarian cancer
- Treatments for ovarian cancer
- Information about living with cancer
- Genetic testing
- Sources of support for those with ovarian cancer (e.g. websites, telephone helpline, group meetings)
- Contact details for the health professionals involved in caring for you
- I do not know

- N/A – all relevant information was covered
- Other

50. Would you like to provide any other information regarding your experience (positive or negative) leading to your diagnosis with ovarian cancer?

Treatments for ovarian cancer: Surgery

The questions in the following sections ask what type of treatments you have had for ovarian cancer. If you have not been offered some of these treatments it may be because they are not suitable for you at this time, or they may not be available in your area.

51. Have you ever had surgery (example: debulking surgery) to treat or control your ovarian cancer?
- Yes
- No ***[skip to Q59]***

52. Have you ever had any of the following? Tick all that apply
- I had chemotherapy before my first surgery
- I had chemotherapy after my first surgery
- I have had only one surgery
- I have had more than one surgery

53. What year did you have your first (or only) surgery for ovarian cancer?

54. How far did you have to travel to have your first (or only) surgery?
- The surgery took place within 30 minutes travelling time from home
- The surgery took place between 30 minutes and 1 hour travelling time from home
- The surgery took place within 1-2 hours travelling time from home
- The surgery took place within 2-4 hours travelling time from home
- The surgery took place over 4 hours travelling time from home

55. Did your first (or only) surgery take place at the same hospital as your chemotherapy?
- Yes
- No
- N/A - I have not had chemotherapy

56. Were you given sufficient information about your first (or only) surgery? (Risks, benefits, recovery, etc.)?
- Yes I was given sufficient information and I understood it
- Yes I was given sufficient information but I did not understand it all
- I do not remember
- No, I was not given sufficient information. If no, what was missing?

57. Did health professionals give you enough time to ask questions about your first (or only) surgery?
- Yes always
- Yes, some of the time

- Not at all
- I do not remember
- I did not have questions

58. Were you able to understand the answers they gave?
- Yes always
- Yes some of the time
- Not at all

Treatments for Ovarian Cancer: Chemotherapy

59. Have you ever had chemotherapy to treat or control your ovarian cancer?
- Yes
- No ***[skip to Q69]***

60. A 'cycle' of chemotherapy is a period of treatment followed by a period of rest (example: you might receive chemotherapy for 1 week followed by 3 weeks with no chemotherapy to make up a 4 week 'cycle'). A 'course' of chemotherapy includes a number of 'cycles' of treatment with a particular drug or combination of drugs (for example: six 4-week cycles is one course of treatment). How many 'courses' of chemotherapy treatment for ovarian cancer have you had?
- 1
- 2
- 3
- 4
- 5
- more than 5

61. What year did you start your first (or only) course of chemotherapy?

62. How far did you have to travel to have your first (or only) course of chemotherapy?
- The chemotherapy took place within 30 minutes travelling time from home
- The chemotherapy took place between 30 minutes and 1 hour travelling time from home
- The chemotherapy took place within 1-2 hours travelling time from home
- The chemotherapy took place within 2-4 hours travelling time from home
- The chemotherapy took place over 4 hours travelling time from home

63. Did you experience any side effects as a result of any of the chemotherapy treatments you have had? Tick all that apply:
- Tiredness or fatigue
- Nausea or vomiting
- Cognitive function ("chemo brain")
- Diarrhea
- Loss of appetite
- Inability to fight infection
- Hair thinning or hair loss

- Dry skin
- Sore mouth
- Neuropathy (tingling or numbness in hands and/or feet)
- Sleep loss
- Metal taste in mouth
- Constipation
- Allergic reaction
- Joint aches or pains
- Lymphedema (swelling of one or more limbs)
- Muscle aches or pains
- Menopausal symptoms
- I did not have any side effects
- I don't know / I cannot remember
- Another side effect

64. Which three side effects did you find most difficult to deal with? Select up to 3 options.

- Tiredness or fatigue
- Nausea or vomiting
- Cognitive function ("chemo brain")
- Diarrhea
- Loss of appetite
- Inability to fight infection
- Hair thinning or hair loss
- Dry skin
- Sore mouth
- Neuropathy (tingling or numbness in hands and/or feet)
- Sleep loss
- Metal taste in mouth
- Constipation
- Allergic reaction
- Joint aches or pains
- Lymphedema (swelling of one or more limbs)
- Muscle aches or pains
- Menopausal symptoms
- No one side effect in particular
- I don't know / I cannot remember
- Another side effect

65. Were health professionals able to help reduce the side effects of chemotherapy, either by giving you other medicines, or advice and information?

- Yes, very much so
- Yes, to some extent
- Not at all
- I did not seek help, or I did not need to seek help about side effects

- I don’t know / I cannot remember

66. Were you given sufficient information about your first (or only) chemotherapy? (Risks, benefits, recovery, etc.)?

- Yes I was given sufficient information and I understood it
- Yes I was given sufficient information but I did not understand it all
- I do not remember
- No, I was not given sufficient information. If no, what was missing?

67. Did health professionals give you enough time to ask questions about your first (or only) chemotherapy?

- Yes always
- Yes, some of the time
- Not at all
- I do not remember
- I did not have questions ***[skip to Q69]***

68. Were you able to understand the answers they gave?

- Yes always
- Yes some of the time
- Not at all
- N/A - I did not have questions

Treatments for Ovarian Cancer: Radiation, Hormone Therapies, Targeted Therapies

This section will be asking about other treatments you may have received for ovarian cancer such as radiation, hormone therapies, and targeted therapies. Targeted therapies are newer treatments that act on processes in the cells (e.g. bevacizumab, olaparib, and niraparib). They do not work for all women with ovarian cancer, so you may not have heard of them.

69. Have you ever been treated with the following? Tick all that apply:

- Radiation therapy
- Anti-angiogenics [e.g. bevacizumab (Avastin)]
- PARP inhibitors [e.g. olaparib (Lynparza), niraparib (Zejula)]
- Aromatase inhibitors [e.g. letrozole (Femara), anastrozole (Arimidex)]
- Anti-estrogens (e.g. tamoxifen)
- Immune therapy (e.g. pembrolizumab)
- None of the above ***[skip to Q74]***
- I don’t remember/I don’t know
- Other (please specify):

70. How far did you have to travel to have the following treatments?

Legend for chart below:

(1) I was able to have this treatment in my home

(2) The treatment took place within 30 minutes travelling time from home

(3) The treatment took place between 30 minutes and 1 hour travelling time from home
(4) The treatment took place within 1-2 hours travelling time from home
(5) The treatment took place within 2-4 hours travelling time from home
(6) The treatment took place over 4 hours travelling time from home
(N/A) I did not receive this type of therapy

- Radiation
- Anti-angiogenics [e.g. bevacizumab (Avastin)]
- PARP inhibitors [e.g. olaparib (Lynparza), niraparib (Zejula)]
- Aromatase inhibitors [e.g. letrozole (Femara), anastrozole (Arimidex)]
- Anti-estrogens (e.g. tamoxifen)
- Immune therapy (e.g. pembrolizumab)

Information about other treatments

71. Were you given enough information about these other types of therapies? (Risks, benefits, recovery, etc.)?

Legend for chart below:
(1) Yes I was given sufficient information and I understood it
(2) Yes I was given sufficient information but I did not understand it all
(3) No, I was not given sufficient information
(4) I do not remember
(N/A) I did not receive this type of therapy

- Radiation
- Anti-angiogenics [e.g. bevacizumab (Avastin)]
- PARP inhibitors [e.g. olaparib (Lynparza), niraparib (Zejula)]
- Aromatase inhibitors [e.g. letrozole (Femara), anastrozole (Arimidex)]
- Anti-estrogens (e.g. tamoxifen)
- Immune therapy (e.g. pembrolizumab)

72. Did health professionals give you enough time to ask questions about other types of therapy?

Legend for chart below:
(1) Yes always
(2) Yes, some of the time
(3) Not at all
(4) I do not remember
(5) I did not have questions
(N/A) I did not receive this type of therapy

- Radiation
- Anti-angiogenics [e.g. bevacizumab (Avastin)]
- PARP inhibitors [e.g. olaparib (Lynparza), niraparib (Zejula)]
- Aromatase inhibitors [e.g. letrozole (Femara), anastrozole (Arimidex)]
- Anti-estrogens (e.g. tamoxifen)
- Immune therapy (e.g. pembrolizumab)

73. Were you able to understand the answers they gave?
Yes always
Yes some of the time
Not at all
N/A

- Radiation
- Anti-angiogenics [e.g. bevacizumab (Avastin)]
- PARP inhibitors [e.g. olaparib (Lynparza), niraparib (Zejula)]
- Aromatase inhibitors [e.g. letrozole (Femara), anastrozole (Arimidex)]
- Anti-estrogens (e.g. tamoxifen)
- Immune therapy (e.g. pembrolizumab)

74. If you were offered a treatment that you had not had before, how much would the following factors affect your choice (if at all)?
0 would mean it was not at all important in terms of your choice.
10 would mean it is the most important factor affecting your choice.

- Possibility of a cure
- Prolonging life
- Method of drug administration (e.g. intravenously by injection, or by mouth)
- Potential side effects
- Number or frequency of cycles in a course of treatment, e.g. once every 3 weeks for six months
- Quality of life
- National approved treatment that most women have
- My doctor recommended it
- Cost

Follow up appointments
75. Approximately how long has it been since you finished your last course of treatment (of any kind)?

- Less than 6 months
- Between 6 months and 1 year
- Between 1 and 2 years
- Between 2 and 3 years
- Between 3 and 4 years
- Between 4 and 5 years
- More than 5 years
- I am currently receiving treatment

76. Currently how often do you have follow up appointments at the hospital?

- Every 3 months
- Every 6 months
- Yearly

- N/A: I don't have follow up appointments/I have been discharged ***[skip to Q79]***
- Other (please specify)

77. What tests or assessments are typically carried out at your follow up appointments? Tick all that apply:

- A physical examination
- A blood test
- A radiological assessment (e.g. scan)
- I am asked about symptoms I might or might not have
- I am asked about side effects of treatments
- I am asked about the emotional and psychological impact of living with the disease and undergoing treatment.
- Other

78. Can you or your family contact appropriate health professionals if you have concerns in between follow up appointments?

- Yes always
- Yes sometimes
- No

Long term side effects

79. Have you been left with long term side effects after treatment has ended? Tick all that apply:
Tiredness or fatigue

- Nausea or vomiting
- Cognitive function ("chemo brain")
- Diarrhea
- Loss of appetite
- Inability to fight infection
- Hair thinning or hair loss
- Dry skin
- Sore mouth
- Neuropathy (tingling or numbness in hands and/or feet)
- Sleep loss
- Metal taste in mouth
- Constipation
- Allergic reaction
- Joint aches or pains
- Lymphedema (swelling of one or more limbs)
- Muscle aches or pains
- Menopausal symptoms
- Lack of interest in sex
- Inability to take part in sexual activity
- Anxiety
- Depression

- I've not had long term side effects *[skip to Q81]*
- I don’t know / I cannot remember *[skip to Q81]*
- I have not yet finished my first treatment *[skip to Q81]*
- Other

80. Which long term side effects have been most difficult for you? Tick up to 3 options:

- Tiredness or fatigue
- Nausea or vomiting
- Cognitive function ("chemo brain")
- Diarrhea
- Loss of appetite
- Inability to fight infection
- Hair thinning or hair loss
- Dry skin
- Sore mouth
- Neuropathy (tingling or numbness in hands and/or feet)
- Sleep loss
- Metal taste in mouth
- Constipation
- Allergic reaction
- Joint aches or pains
- Lymphedema (swelling of one or more limbs)
- Muscle aches or pains
- Menopausal symptoms
- Lack of interest in sex
- Inability to take part in sexual activity
- Anxiety
- Depression
- No one side effect in particular
- I don’t know / I cannot remember
- Other

Second opinions

81. Have you ever wanted to get a 'second opinion' from another ovarian cancer doctor?

- Yes
- No *[skip to Q84]*

82. Have you ever asked for a second opinion from another ovarian cancer doctor?

- Yes
- No. If no, why not? *[skip to Q84]*

83. Were you able to get a second opinion from another ovarian cancer doctor?

- Yes
- No. If no, why not?

Clinical Trials

Clinical trials are medical research studies that test potential new treatments and / or compare them to existing treatments.

84. At any point since diagnosis, have you been asked if you would like to join a clinical trial?

- Yes
- I was not asked, but I asked about trials
- No
- I do not know / I cannot remember

85. Were you given information to help you decide whether you wanted to take part in the clinical trial being offered?

- I was given information I understood
- I was given information, but I did not understand it
- I was not given information
- I do not know / I cannot remember

86. Was there a clinical trial you were eligible to join?

- Yes
- No
- I do not know / I cannot remember

87. Did you take part in the clinical trial being offered?

- Yes
- No
- I wanted to take part but then was not able to. Please explain:

Future clinical trials

88. In future, if you were offered a clinical trial, would you be prepared to go to another hospital so you could take part?

- Yes
- No, I do not want to participate in clinical trials at another hospital
- No, I do not want to participate in clinical trials at all

89. How important might the following factors be for you in terms of deciding to join a clinical trial?

0 would indicate it was not at all important, 10 would indicate it was very important.

- The possibility of a cure
- The possibility to extend my life
- Extra tests and check ups
- Advancement of ovarian cancer treatments
- Potentially helping other women in the future

90. What might prevent you from joining a clinical trial?

0 would mean it was not important at all, 10 would mean a very important reason.

- Distance to travel
- I may not personally benefit
- Number of hospital visits
- Impact on friends and family
- Potential side effects
- Concern about safety of drugs in the Trial
- Cost
- Worried I will get a placebo and not receive adequate treatment
- I worry I will be locked into participating

Support needs

Being told that you have ovarian cancer is a life changing experience. It can be extremely challenging for some women to come to terms with their diagnosis and cope with difficult emotions, practical and financial support needs. This section explores these challenges.

91. Have there been particular times when you have felt in need of emotional support? Tick all that apply:

- At the point of diagnosis
- After the initial treatment was over
- When the cancer returned
- When you were told the cancer is not curable
- I did not feel in need of emotional support
- Other

92. Are there particular issues you have faced? Tick all that apply:

- Fear of your cancer coming back
- Fear that treatment will not work
- Fear of dying
- Getting your life back on track after treatment
- Issues relating to family and friends
- Feelings of isolation
- Feeling unable to talk to others about your illness
- Issues relating to body image
- Regaining sexual intimacy with a partner
- Loss of fertility
- Coping with treatment-induced menopause
- None in particular
- Other:

93. Which issue have you found the most challenging? Select up to 3 options:

- Fear of your cancer coming back
- Fear that treatment will not work
- Fear of dying

- Getting your life back on track after treatment
- Issues relating to family and friends
- Feelings of isolation
- Feeling unable to talk to others about your illness
- Issues relating to body image
- Regaining sexual intimacy with a partner
- Loss of fertility
- Coping with treatment-induced menopause
- Not one in particular
- Other

Getting emotional support

94. Did you seek out emotional support, or were you offered emotional support by healthcare professionals? Tick all that apply:

- Yes, I sought help from a health professional
- Yes, I was offered help from a health professional
- No, I neither sought help, nor was I offered help

95. Which groups or people have given you the most emotional support? Select up to 3 options:

- A member of my healthcare team
- A social worker
- A counsellor or psychotherapist
- A psychologist or psychiatrist
- A faith leader or group
- A support group
- Other women or another woman with ovarian cancer
- A telephone helpline
- An online discussion board (e.g. OVdialogue)
- Social media (e.g. Facebook groups, Instagram accounts)
- Family or friends
- A health charity or organization (e.g. Ovarian Cancer Canada)
- No one in particular
- Other (please specify)

Meeting women with ovarian cancer

96. Have you ever met or talked to another woman, or group of women, who have had ovarian cancer? Tick all that apply:

- Yes, in person
- Yes, online (Facebook, chat room, forum)
- Yes, on the telephone
- No

97. Would you like to be able to connect with other women who have or have had ovarian cancer?

- Yes
- No ***[skip to Q99]***

- I'm not sure

98. What would be your preferred way to meet with these other women?
- In person, one on one
- In person, in a group setting
- Online, one on one
- Online, in a group setting
- Telephone
- N/A - I would prefer not to meet other women with ovarian cancer at this time

More about emotional support

99. How much do you agree with the following statements about meeting women with ovarian cancer?

0 would be totally disagree, 5 would be neither agree or disagree, and 10 would be totally agree.
- It helped or helps me understand that I am not alone in facing the disease
- I find it easier to talk to women who have been through the same experience
- It feels positive to share stories with each other
- I find out information about possible treatments or sources of support
- We raise awareness about the disease together
- I find out tips for living with the disease
- I feel more confident in approaching my doctor to ask certain questions
- I find it hard when someone I know who has ovarian cancer has a recurrence or dies
- It makes me more anxious that my cancer will come back or become untreatable
- I find/found it hard to talk to other women who have ovarian cancer
- I'd rather live my life away from cancer, so prefer not to be in touch with other women with ovarian cancer

Practical support needs

100. Which, if any, of the following forms of practical support do you feel you need, or have you needed because of your diagnosis and treatment for ovarian cancer?

Tick all that apply:
- Help with daily chores at home (e.g. shopping, cleaning, gardening, preparing food)
- Help with personal care (e.g. getting dressed or undressed, getting washed, wound care)
- Home adaptations (e.g. using a wheelchair, handrails)
- Help with caring for dependents (eg parents, siblings, children)
- Help with transport, including travel to and from hospital
- Financial support
- I have not needed any practical support
- Other

101. Which, if any, of the following forms of practical support have you received following your diagnosis and treatment for ovarian cancer? Tick all that apply:
- Help with daily chores at home (e.g. shopping, cleaning, gardening, preparing food)
- Help with personal care (e.g. getting dressed or undressed, getting washed, wound care)

- Home adaptations (e.g. using a wheelchair, handrails)
- Help with caring for dependents (e.g. parents, siblings, children)
- Help with transport, including travel to and from hospital
- Financial support
- I have not received any practical support
- Other:

102. Which group or person has given you the most practical support? Select up to 3 options:
- Family member/s
- Friend/s
- A cancer support organization (e.g. Ovarian Cancer Canada)
- A faith-based organization
- Other women or another woman with ovarian cancer
- A caregiver
- No one in particular
- Other

Financial support needs

103. Has having a diagnosis of ovarian cancer impacted on your financial situation?
- Yes to a great extent
- Yes to some extent
- Not at all

104. In what way/s have your finances been affected by your diagnosis?

Information support needs

105. Since being diagnosed, have you felt in need of information about ovarian cancer? Tick all that apply:
- Ovarian cancer in general
- Treatments for ovarian cancer
- Coping with long term side effects
- Clinical trials
- Genetic testing
- Living with ovarian cancer
- Reducing anxiety
- Symptoms that might indicate recurrence
- Survival rates
- Managing ovarian cancer that can no longer be treated
- How to talk to family and friends
- Nutrition and exercise
- Spiritual support/mindfulness/meditation
- I have not needed information
- Other

106. Have you found the information you needed?
- Yes always
- Yes to some extent
- Just a little information
- Not at all

107. What have been your most important sources of information? Tick up to 3 choices:
- Your doctor
- Your nurse
- Another health professional
- Ovarian Cancer Canada
- An ovarian cancer support group (online or face to face)
- Other women who have had ovarian cancer
- Other (please specify):

Final questions

108. What does having a good quality of life mean to you at this point in time?
Please rate the following statements, with 0 as not at all important in terms of your quality of life, and 10 most important for your quality of life. Try to make sure that the thing that is most important is given the highest score of all your ratings.
- The extent to which you feel physically well
- The extent to which you feel mentally well
- The extent to which you have a sense of purpose in life
- The extent to which you feel you have control of what is happening in your life
- Your ability to work
- The ability to provide for your family
- Your ability to socialize
- Your ability to engage in hobbies and activities
- The extent to which you have a positive self image
- The quality of your sexual health and function after ovarian cancer (including pain free intercourse, return of libido)
- Your capacity to enjoy positive sexual relationships (including self image, relationship with partner)
- Your capacity to feel joy

109. How would you rate your quality of life today, on a scale of 0 to 10, with 10 being the best possible score. Move the slider along to change the display.
I rate my quality of life as: 0 to 10

How can Ovarian Cancer Canada advocate on behalf of women affected by ovarian cancer?

110. What do you regard as the most important priorities for improving outcomes of women with or at risk of ovarian cancer in Canada? Tick up to 3 options:
- Improving access to genetic testing
- Prevention of ovarian cancer

- Raising awareness of the symptoms of ovarian cancer
- Finding an effective ovarian cancer screening tool
- Improving diagnostic tests
- Reducing delays in diagnosis
- Ensuring access to gynecological cancer specialist doctors
- Development of new surgical techniques
- Ensuring access to existing drug treatments
- Ensuring access to clinical trials
- Development of new drug treatments
- Ensuring access to new drug treatments

111. Did we miss anything that would be important for us to advocate about?

112. Is there anything else you would like to share about your experience living with ovarian cancer that was not captured in this survey?

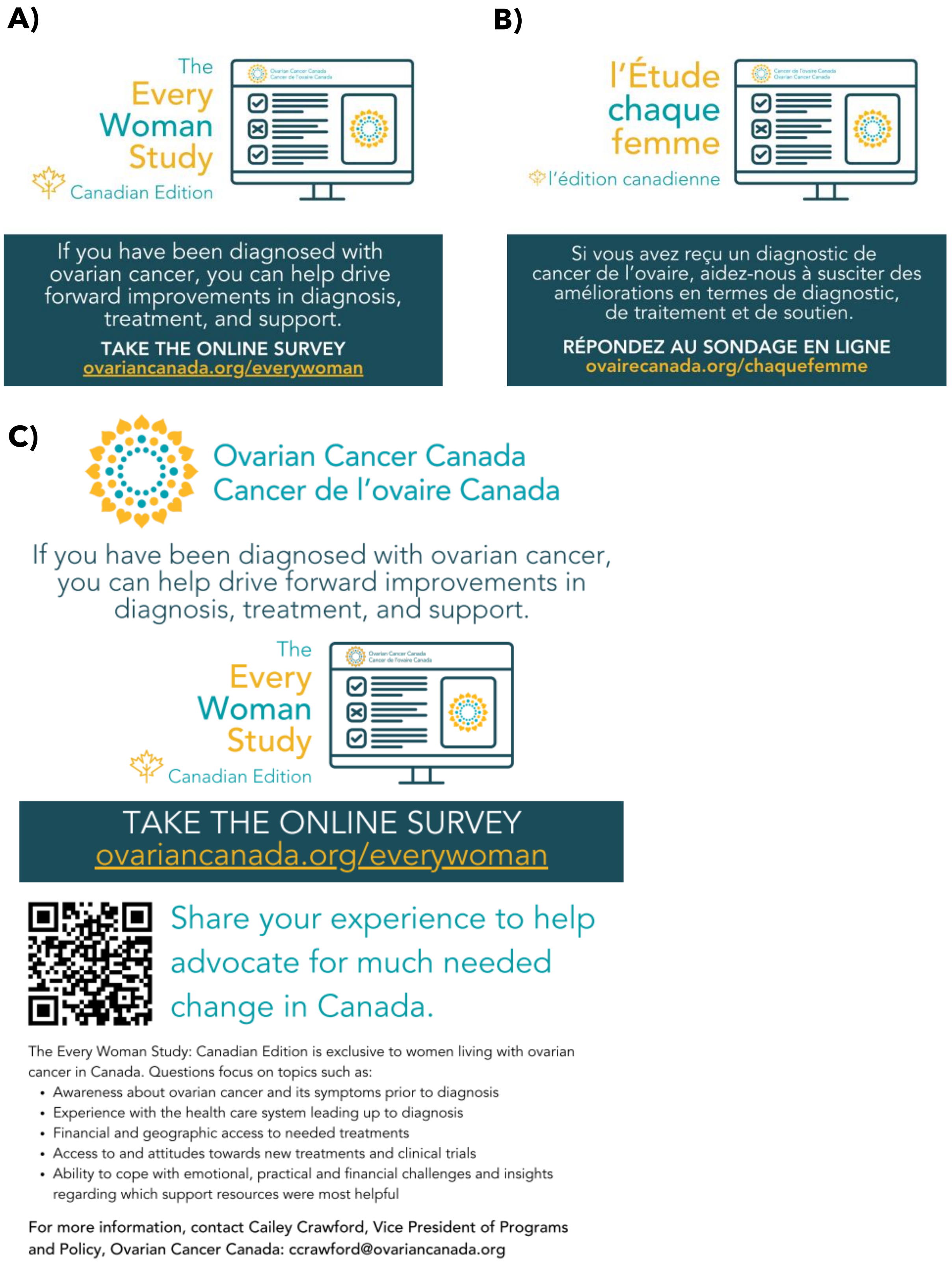

Figure S1. Recruitment materials shared through social media and Ovarian Cancer Canada's collaborative networks.

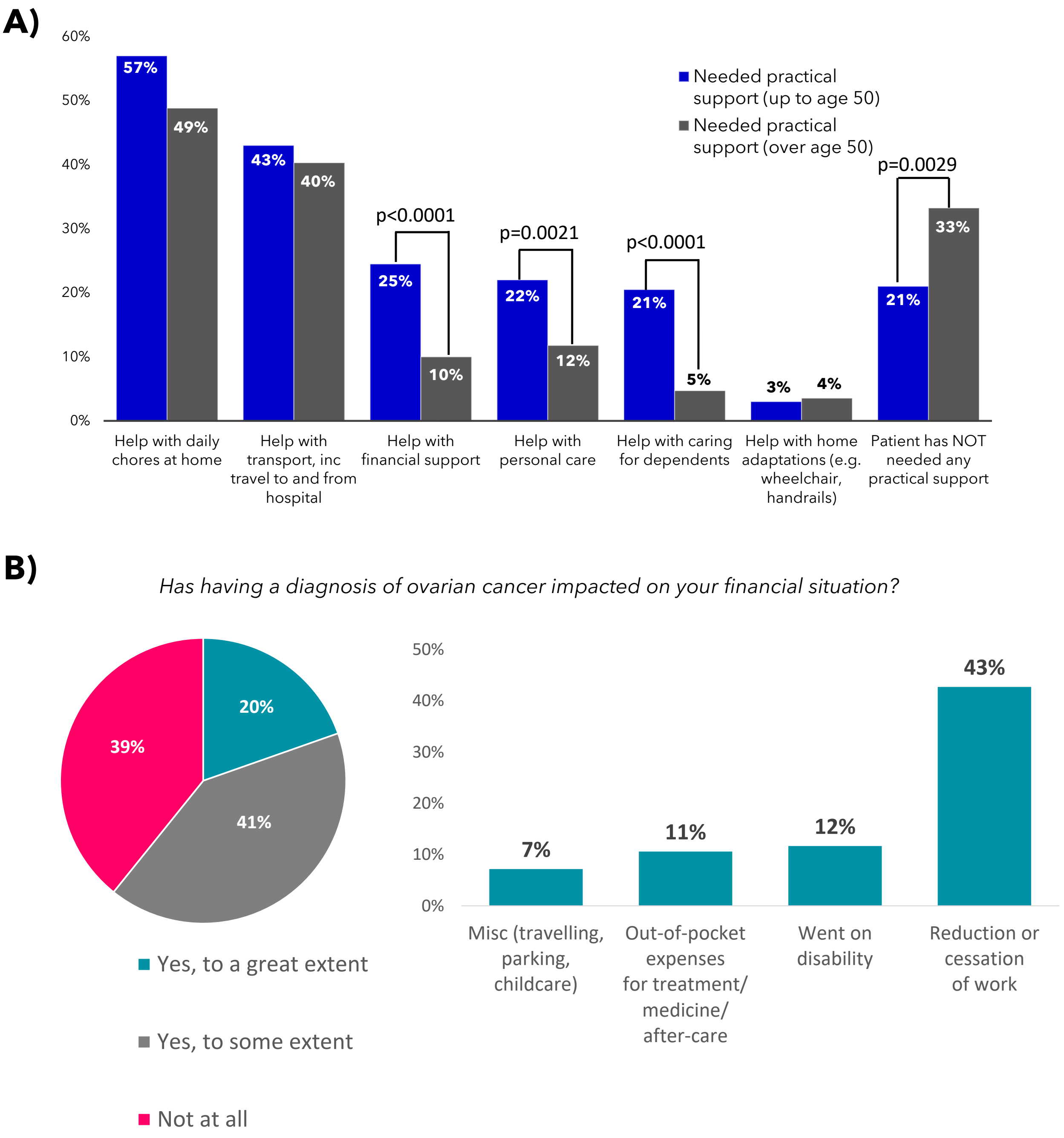

Figure S2. Practical impacts of ovarian cancer.

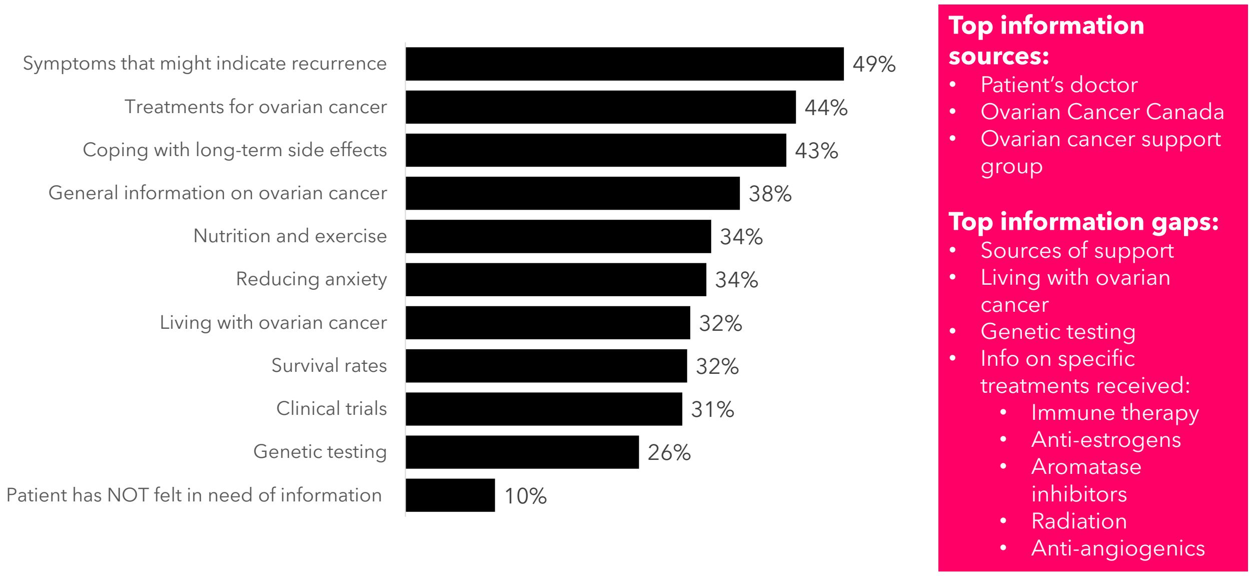

Figure S3. Patient-reported information needs.

Table S1. A snapshot of regional differences (top 5).

	ON (*n* = 221)	QC (*n* = 100)	BC (*n* = 83)	AB (*n* = 70)	SK (*n* = 33)
**Age at diagnosis**	*n = 218*	*n = 97*	*n = 77*	*n = 68*	*n = 31*
≤50	80 (37%)	40 (41%)	18 (23%)	29 (43%)	14 (45%)
>50	138 (63%)	57 (59%)	59 (77%)	39 (57%)	17 (55%)
**First healthcare provider**	*n = 182*	*n = 84*	*n = 66*	*n = 61*	*n = 30*
Family doctor	131 (72%)	44 **(52%)**	46 (70%)	45 (74%)	24 **(80%)**
ER/urgent care	33 (18%)	29 **(35%)**	17 (26%)	13 (21%)	2 **(7%)**
**Tests ordered**	*n = 181*	*n = 85*	*n = 64*	*n = 59*	*n = 30*
Yes	141 (78%)	64 (75%)	53 (83%)	46 (78%)	22 (73%)
No, but referral made	19 (10%)	3 (4%)	5 (8%)	2 (3%)	4 (13%)
No tests ordered/no referrals	21 (12%)	18 (21%)	6 (9%)	11 (19%)	4 (13%)
**Time to diagnosis**	*n = 212*	*n = 98*	*n = 76*	*n = 67*	*n = 32*
<1 month	60 (28%)	49 **(50%)**	30 (39%)	20 (30%)	4 **(13%)**
1-3 months	69 (33%)	26 (27%)	23 (30%)	18 (27%)	7 (22%)
>3 months	83 (39%)	23 **(23%)**	23 (30%)	29 (43%)	20 **(63%)**
**Year of Diagnosis**	*n = 218*	*n = 99*	*n = 83*	*n = 67*	*n = 32*
Up to 2010	29 (13%)	12 (12%)	12 (14%)	11 (16%)	10 (31%)
2011-2015	54 (24%)	25 (25%)	19 (23%)	15 (22%)	8 (25%)
2016-2020	135 (61%)	62 (63%)	52 (63%)	41 (61%)	14 (44%)
2011-2020	*87%*	*88%*	*86%*	*84%*	*69%*
**Type of OC**	*n = 216*	*n = 100*	*n = 79*	*n = 68*	*n = 31*
High-grade serous	106 (49%)	48 (48%)	47 (59%)	26 (38%)	9 (29%)
Endometrioid/clear cell	37 (17%)	8 (8%)	10 (13%)	10 (15%)	3 (10%)
Low-grade serous	10 (5%)	9 (9%)	3 (4%)	5 (7%)	3 (10%)
Mucinous	5 (2%)	1 (1%)	2 (3%)	2 (3%)	0
Non-epithelial cancer	21 (10%)	5 (5%)	3 (4%)	7 (10%)	5 (16%)
Borderline tumour	6 (3%)	3 (3%)	2 (3%)	8 (12%)	1 (3%)
Mixed/other	10 (5%)	7 (7%)	2 (3%)	2 (3%)	1 (3%)
Don't know	21 (10%)	19 (19%)	10 (13%)	8 (12%)	9 (29%)
**Stage of Diagnosis**	*n = 219*	*n = 100*	*n = 79*	*n = 68*	*n = 30*
I	50 (23%)	18 (18%)	11 (14%)	18 (26%)	10 (33%)
II	35 (16%)	10 (10%)	14 (18%)	10 (15%)	4 (13%)
III	103 (47%)	58 (58%)	42 (53%)	32 (47%)	14 (47%)
IV	26 (12%)	10 (10%)	8 (10%)	6 (9%)	2 (7%)
III/IV	*59%*	*68%*	*63%*	*56%*	*54%*
Unsure	5 (2%)	4 (4%)	4 (5%)	2 (3%)	0
**Post-diagnosis genetic testing offered***	*n = 197*	*n = 95*	*n = 73*	*n = 67*	*n = 30*
Yes	148 (75%)	76 (80%)	63 **(86%)**	47 (70%)	14 **(47%)**
Not offered	47 (24%)	18 (19%)	9 (12%)	18 (27%)	14 (47%)
Not interested	2 (1%)	1 (1%)	1 (1%)	2 (3%)	2 (7%)
**Clinical trial offered**	*n = 177*	*n = 85*	*n = 73*	*n = 57*	*n = 30*
Yes	49 (28%)	33 **(39%)**	17 (23%)	10 (18%)	2 **(7%)**
No	123 (69%)	51 (60%)	53 (73%)	45 (79%)	26 (87%)
Don't remember	5 (3%)	1 (1%)	3 (4%)	2 (4%)	2 (7%)

***excluded: tested before diagnosis, not sure, timing not provided**

Table S2. Different types of ovarian cancer awareness prior to diagnosis.

	General OC Awareness = Yes*	General OC Symptom Awareness = Yes**	Matched Symptom Awareness = Yes***
Full cohort	186/537 (35%)	240/531 (45%)	190/472 (40%)
Region			
BC	29/77 (38%)	30/75 (40%)	
AB	23/67 (34%)	32/68 (47%)	
SK	13/32 (41%)	18/31 (58%)	
MB	9/16 (56%)	9/16 (56%)	---
ON	84/212 (40%)	105/210 (50%)	
QC	20/100 **(20%)**	32/99 **(32%)**	
NS	4/19 (21%)	9/18 (50%)	
Urban vs. rural			
Urban	161/461 (35%)	210/456 (46%)	---
Rural	26/71 (37%)	29/70 (41%)	
Age at diagnosis			
≤50	60/194 (31%)	89/191 (47%)	---
>50	127/337 (38%)	147/333 (44%)	
Family history of ovarian cancer			
Yes (N=120)	57 (48%)	54 (45%)	
No (N=407)	125 (31%)	181 (44%)	---
p-value	**0.0006**	1.000	

* includes *"I knew a lot about it"* (5% of full cohort) and *"I knew a bit about it"* (30% of full cohort). ** prior knowledge of at least one OC symptom; includes persistent bloating (28% of cohort), abdominal pain (27%), menstrual irregularities (21%), fatigue (17%), unexplained weight loss/gain (15%), changes in bowel habits (14%), difficulty eating (10%), urinary symptoms (9%). *** prior knowledge of at least one OC symptom <u>that the individual experienced</u>. OC = ovarian cancer.

Table S3. Symptoms <u>experienced</u> by stage, type and age.

A) Symptoms experienced by stage at diagnosis

# **Who Experienced Symptom (Select All That Apply)**	**Full Cohort (n = 537)***	**Stage I/II (n = 202)**	**Stage III/IV (n = 316)**	***p*-Value (I/II vs. III/IV)**
Persistent bloating	320 (60%)	106 (52%)	205 **(65%)**	***p* = 0.0033**
Abdominal pain	311 (58%)	116 (57%)	184 (58%)	*p* = 0.8224
Extreme/persistent fatigue	243 (45%)	83 (41%)	153 (48%)	*p* = 0.1189
Urinary symptoms	203 (38%)	76 (38%)	124 (39%)	*p* = 0.8198
Change in bowel habits	193 (36%)	53 (26%)	136 **(43%)**	***p* = 0.0001**
Difficulty eating	167 (31%)	46 (23%)	116 **(37%)**	***p* = 0.0008**
Unexplained weight loss/gain	165 (31%)	55 (27%)	102 (32%)	*p* = 0.2265
Menstrual irregularities	124 (23%)	61 **(30%)**	55 (17%)	***p* = 0.0005**

*includes those diagnosed at unknown/unspecified stage

B) Symptoms experienced by ovarian cancer type

# **Who Consider Symptom Most Concerning (Select One)**	**High-Grade Serous Cancer (n = 245)**	**Other Epithelial Cancer (n = 112)**	**Combined Epithelial Cancer (n = 357)**	**Non-Epithelial Cancer (n = 43)**
Persistent bloating	153 (62%)	67 (60%)	220 (62%)	21 (49%)
Abdominal pain	140 (57%)	72 (64%)	212 (59%)	27 (63%)
Extreme/persistent fatigue	113 (46%)	50 (45%)	163 (46%)	20 (47%)
Urinary symptoms	91 (37%)	52 (46%)	143 (40%)	14 (33%)
Change in bowel habits	93 (38%)	44 (39%)	137 **(38%)**[#]	7 **(16%)**
Difficulty eating	78 (32%)	36 (32%)	114 (32%)	12 (28%)
Unexplained weight loss/gain	71 (29%)	39 (35%)	110 (31%)	13 (30%)
Menstrual irregularities	36 (15%)	38 (34%)	74 **(21%)**	24 **(56%)**[#]

[#]Statistically significant comparison by type.

C) Symptoms experienced among respondents diagnosed at stage I/II

# Who Experienced Symptom	All Types (*n* = 202)		High-Grade Serous Cancer (*n* = 51)			Other Epithelial Cancer (*n* = 62)*			Non-Epithelial Cancer (*n* = 33)		
	≤50 (89)	>50 (112)	All ages	≤50 (9)	>50 (41)	All ages	≤50 (30)	>50 (32)	All ages	≤50 (20)	>50 (13)
Persistent bloating	45 (51%)	60 (54%)	26 (51%)	4 (44%)	21 (51%)	31 (50%)	13 (43%)	18 (56%)	17 (52%)	10 (50%)	7 (54%)
Abdominal pain	61 **(69%)****	55 **(49%)**	25 (49%)	4 (44%)	21 (51%)	39 (63%)	21 (70%)	18 (56%)	20 (61%)	14 (70%)	6 (46%)
Extreme/ persistent fatigue	45 **(51%)****	38 **(34%)**	17 (33%)	5 (56%)	12 (29%)	26 (42%)	14 (47%)	12 (38%)	13 (39%)	8 (40%)	5 (38%)
Urinary symptoms	34 (38%)	42 (38%)	19 (37%)	3 (33%)	16 (39%)	25 (40%)	12 (40%)	13 (41%)	11 (33%)	6 (30%)	5 (38%)
Change in bowel habits	27 (30%)	25 (22%)	12 (24%)	3 (33%)	8 (20%)	20 (32%)	12 (40%)	8 (25%)	5 (15%)	4 (20%)	1 (8%)
Difficulty eating	25 (28%)	20 (18%)	9 (18%)	2 (22%)	6 (15%)	17 (27%)	12 **(40%)**[&]	5 **(16%)**	8 (24%)	5 (25%)	3 (23%)
Unexplained weight loss/gain	31 **(35%)****	23 **(21%)**	12 (24%)	4 (44%)	7 (17%)	20 (32%)	13 (43%)	7 (22%)	10 (30%)	7 (35%)	3 (23%)
Menstrual irregularities	38 **(43%)****	27 **(24%)**	10 (20%)	2 (22%)	7 (17%)	18 (29%)	11 (37%)	7 (22%)	20 **(61%)**[#]	14 (70%)	6 (46%)

* clear cell, endometrioid and low-grade serous included in analysis. ** Statistically significant comparisons by age within stage I/II (all types). [&] Statistically significant comparison by age within stage I/II (within type). [#] Statistically significant comparison by type within stage I/II [menstrual irregularities increased in non-epithelial vs. combined epithelial (24%)]

D) Symptoms experienced among respondents diagnosed at stage III/IV

# Who Experienced Symptom	All Types (*n* = 316)		High-Grade Serous Cancer (*n* = 193)			Other Epithelial Cancer (*n* = 48)*			Non-Epithelial Cancer (*n* = 9)		
	≤50 (93)	>50 (217)	All ages	≤50 (40)	>50 (149)	All ages	≤50 (25)	>50 (22)	All ages	≤50 (6)	>50 (3)
Persistent bloating	66 (71%)	133 (61%)	127 (66%)	29 (73%)	94 (63%)	34 (71%)	16 (64%)	17 (77%)	4 (44%)	---	---
Abdominal pain	64 **(69%)****	115 **(53%)**	115 (60%)	27 (68%)	85 (57%)	32 (67%)	18 (72%)	13 (59%)	6 (67%)	---	---
Extreme/persistent fatigue	50 (54%)	97 (45%)	96 (50%)	23 (58%)	69 (46%)	23 (48%)	10 (40%)	12 (55%)	6 (67%)	---	---
Urinary symptoms	44 **(47%)****	75 **(35%)**	72 (37%)	18 (45%)	51 (34%)	26 (54%)	14 (56%)	11 (50%)	3 (33%)	---	---
Change in bowel habits	40 (43%)	94 (43%)	81 (42%)	17 (43%)	62 (42%)	23 (48%)	12 (48%)	11 (50%)	2 (22%)	---	---
Difficulty eating	44 **(47%)****	69 **(32%)**	69 (36%)	20 **(50%)**[&]	47 **(32%)**	18 (38%)	11 (44%)	6 (27%)	3 (33%)	---	---
Unexplained weight loss/gain	40 **(43%)****	58 **(27%)**	59 (31%)	17 **(43%)**[&]	39 **(26%)**	17 (35%)	11 (44%)	5 (23%)	3 (33%)	---	---
Menstrual irregularities	39 **(42%)****	23 **(11%)**	26 **(13%)**	15 **(38%)**[&]	11 **(7%)**	19 **(40%)**[#]	13 (52%)	6 (27%)	3 **(33%)**[#]	---	---

* clear cell, endometrioid and low-grade serous included in analysis. ** Statistically significant comparisons by age within stage III/IV (all types). [&] Statistically significant comparison by age within stage III/IV (within type). [#] Statistically significant comparison by type within stage III/IV [menstrual irregularities decreased in high-grade serous vs. other epithelial/non-epithelial combined (39%)]

Table S4. Most concerning symptom by stage, type and age.

A) Most concerning symptom by stage at diagnosis (irrespective of ovarian cancer type or age)

# **Who Consider Symptom Most Concerning (Select One)**	**Full Cohort (n = 466)**	**Stage I/II (n = 176)**	**Stage III/IV (n = 276)**	**p-Value (I/II vs. III/IV)**
Persistent bloating	86 (18%)	22 **(13%)**	62 **(22%)**	**0.0164**
Abdominal pain	131 (28%)	51 (29%)	75 (27%)	0.6438
Extreme/persistent fatigue	29 (6%)	12 (7%)	16 (6%)	0.6720
Urinary symptoms	32 (7%)	15 (9%)	16 (6%)	0.2285
Change in bowel habits	30 (6%)	5 **(3%)**	25 **(9%)**	**0.0127**
Difficulty eating	15 (3%)	4 (2%)	11 (4%)	0.2408
Unexplained weight loss/gain	9 (2%)	2 (1%)	7 (3%)	0.1599
Menstrual irregularities	37 (8%)	23 **(13%)**	12 **(4%)**	**0.0004**

B) Most concerning symptom by ovarian cancer type

# **Who Consider Symptom Most Concerning (Select One)**	**High-Grade Serous Cancer (n = 211)**	**Other Epithelial Cancer (n = 101)**	**Combined Epithelial Cancer (n = 312)**	**Non-Epithelial Cancer (n = 39)**
Persistent bloating	49 **(23%)**[#]	13 (13%)	62 (20%)	2 (5%)
Abdominal pain	56 (27%)	39 (39%)	95 (30%)	14 (36%)
Extreme/persistent fatigue	16 (8%)	9 (9%)	25 (8%)	0
Urinary symptoms	16 (8%)	6 (6%)	22 (7%)	2 (5%)
Change in bowel habits	16 (8%)	3 (3%)	19 (6%)	0
Difficulty eating	6 (3%)	3 (3%)	9 (3%)	0
Unexplained weight loss/gain	2 (1%)	2 (2%)	4 (1%)	0
Menstrual irregularities	8 (4%)	7 (7%)	15 **(5%)**	11 **(28%)**[#]

[#] Statistically significant comparison by type.

C) Most concerning symptom among respondents diagnosed at stage I/II

# **Who Consider Symptom Most Concerning**	**All Types (*n*=176)**		**High-Grade Serous Cancer (*n*=40)**			**Other Epithelial Cancer (*n*=55)***			**Non-Epithelial Cancer (*n* = 31)**		
	≤50 (85)	>50 (90)	All ages	≤50 (7)	>50 (32)	All ages	≤50 (29)	>50 (26)	All ages	≤50 (19)	>50 (12)
Persistent bloating	8 (9%)	13 (14%)	8 (20%)	1	6 (19%)	6 (11%)	2 (7%)	4 (15%)	2 (6%)	1 (5%)	1 (8%)
Abdominal pain	29 (34%)	22 (24%)	9 (23%)	0	9 (28%)	22 (40%)	13 (45%)	9 (35%)	9 (29%)	7 (37%)	2 (17%)
Extreme/persistent fatigue	4 (5%)	8 (9%)	3 (8%)	1	2 (6%)	6 (11%)	2 (7%)	4 (15%)	0	---	---
Urinary symptoms	6 (7%)	9 (10%)	5 (13%)	1	4 (13%)	3 (5%)	1 (3%)	2 (8%)	2 (6%)	0	2 (17%)
Change in bowel habits	1 (1%)	4 (4%)	1 (3%)	0	1 (3%)	0	---	---	0	---	---
Difficulty eating	3 (4%)	1 (1%)	0	---	---	2 (4%)	1 (3%)	1 (4%)	0	---	---
Unexplained weight loss/gain	1 (1%)	1 (1%)	0	---	---	1 (2%)	1 (3%)	0	0	---	---
Menstrual irregularities	13 (15%)	10 (11%)	4 **(10%)**	1	3 (9%)	4 **(7%)**	2 (7%)	2 (8%)	9 **(29%)**#	7 (37%)	2 (17%)

* clear cell, endometrioid and low-grade serous included in analysis. # Statistically significant comparison by type within stage I/II [menstrual irregularities increased in non-epithelial vs. epithelial (8% combined)]. *Note: no statistically significant comparisons by age within stage I/II (all types or within type)*

D) Most concerning symptom among respondents diagnosed at stage III/IV

# **Who Consider Symptom Most Concerning**	**All Types (*n* = 276)**		**High-Grade Serous Cancer (*n* = 171)**			**Other Epithelial Cancer (*n* = 44)***			**Non-Epithelial Cancer (*n* = 7)**		
	≤50 (87)	>50 (183)	All ages	≤50 (36)	>50 (131)	All ages	≤50 (24)	>50 (19)	All ages	≤50 (5)	>50 (2)
Persistent bloating	19 (22%)	41 (22%)	41 (24%)	7 (19%)	32 (24%)	6 (14%)	4 (17%)	2 (11%)	0	---	---
Abdominal pain	28 (32%)	46 (25%)	47 (27%)	12 (33%)	34 (26%)	17 (39%)	11 (46%)	6 (32%)	4 (57%)	---	---
Extreme/persistent fatigue	5 (6%)	10 (5%)	13 (8%)	3 (8%)	10 (8%)	3 (7%)	2 (8%)	0	0	---	---
Urinary symptoms	4 (5%)	12 (7%)	11 (6%)	2 (6%)	9 (7%)	2 (5%)	1 (4%)	1 (5%)	0	---	---
Change in bowel habits	2 (2%)	23 **(13%)****	15 (9%)	1 (3%)	14 (11%)	3 (7%)	0	3 **(16%)**[&]	0	---	---
Difficulty eating	3 (3%)	7 (4%)	6 (4%)	2 (6%)	4 (3%)	1 (2%)	1 (4%)	0	0	---	---
Unexplained weight loss/gain	2 (2%)	5 (3%)	2 (1%)	1 (3%)	1 (1%)	1 (2%)	0	1 (5%)	0	---	---
Menstrual irregularities	8 **(9%)****	4 (2%)	4 (2%)	2 (6%)	2 (2%)	3 (7%)	2 (8%)	1 (5%)	2 **(29%)**[#]	---	---

* clear cell, endometrioid and low-grade serous included in analysis. ** Statistically significant comparisons by age within stage III/IV (all types). [&] Statistically significant comparison by age within stage III/IV (within type). [#] Statistically significant comparison by type within stage III/IV [menstrual irregularities increased in non-epithelial vs. epithelial (3% combined)].

Table S5. Differences between symptomatic and asymptomatic respondents.

Variable		Symptomatic (*n* = 479)[1]	Asymptomatic (*n* = 58)	*p*-Value
**Age at diagnosis**		*n = 472*		
	≤50	181 (38%)	14 (24%)	**0.0368**
	>50	291 **(62%)**	44 **(76%)**	
**Time to diagnosis**[2]		*n = 473*	*n = 57*	
	<1 month	148 **(31%)**	29 **(51%)**	**0.0025**
	1-3 months	141 (30%)	19 (33%)	0.6419
	>3 months	184 **(39%)**	9 **(16%)**	**0.0007**
**Stage at diagnosis**		*n = 476*	*n = 57*	
	I	104 (22%)	17 (30%)	0.1745
	II	77 (16%)	4 (7%)	0.0727
	III	241 (51%)	21 (37%)	0.0459
	IV	43 (9%)	11 (19%)	0.0178
	III/IV	*284 (60%)*	*32 (56%)*	*0.5612*
	Unsure	11 (2%)	4 (7%)	0.0234
**Ovarian cancer type**		*n = 474*		
	High-grade serous	218 (46%)	27 (47%)	0.8854
	Endometrioid or clear cell	67 (14%)	9 (16%)	0.6809
	Low-grade serous	34 (7%)	2 (3%)	0.2460
	Mucinous	11 (2%)	0	0.2776
	Non-epithelial cancer	40 (8%)	3 (5%)	0.4182
	Mixed	5 (1%)	1 (2%)	0.4928
	Borderline tumour	21 (4%)	2 (3%)	0.7103
	Other	17 (4%)	4 (7%)	0.2896
	Don't know/can't remember	61 (13%)	10 (17%)	0.3996

[1] includes all respondents who experienced one or more symptoms, regardless of whether initiated consultation with a healthcare provider to investigate their symptoms. [2] from first visit with a healthcare professional, regardless of what led to first appointment.

Table S6. Differences between respondents who did vs. did not consult a healthcare provider about their symptoms.

Variable	Number (%) in a Specific Category Who Consulted	Proportion of Those Who Consulted (*n* = 400)	Proportion of Those Who Did Not Consult (*n* = 65)*	*p*-Value (Did vs. Did Not)
**Concern level about symptoms**				
Very or fairly concerned (*n* = 295)	283 (96%)	**71%**	12 **(18%)**	**<0.0001**
Not very/not at all concerned (*n* = 170)	117 (69%)	29%	53 (82%)	
p-value (% consulted by category)	**<0.0001**	---	---	
**Most concerning symptom**				
Abdominal pain (*n* = 131)	123 (94%)	**31%**	8 **(12%)**	**0.0016**
Persistent bloating (*n* = 86)	78 (91%)	20%	8 (12%)	0.1268
Menstrual irregularities (*n* = 37)	37 (100%)	**9%**	**0%**	**0.0119**
Urinary symptoms (*n* = 32)	29 (91%)	7%	3 (5%)	0.5507
Change in bowel habits (*n* = 30)	23 (77%)	6%	7 (11%)	0.1352
Extreme/persistent fatigue (*n* = 28)	21 (75%)	5%	7 (11%)	0.0560
Difficulty eating (*n* = 15)	12 (80%)	3%	3 (5%)	0.4016
Unexplained weight loss/gain (*n* = 9)	8 (89%)	2%	1 (2%)	1.000
None in particular (*n* = 31)	7 (23%)	**2%**	24 **(37%)**	**<0.0001**
**Age at diagnosis**				
≤50 (*n* = 181)	166 (92%)	**42%**	15 (23%)	**0.0037**
>50 (*n* = 277)	227 (82%)	57%	50 **(77%)**	
p-value (% consulted by category)	**0.0026**	---	---	
**Family history of ovarian cancer**				
Yes (*n* = 99)	86 (87%)	22%	13 (20%)	0.7171
No (*n* = 355)	303 (85%)	76%	52 (80%)	
p-value (% consulted by category)	0.6183	---	---	
**Family history of ovarian/breast cancer**				
Both (*n* = 72)	61 (85%)	15%	11 (17%)	0.6780
Ovarian only (*n* = 27)	25 (93%)	6%	2 (3%)	0.3290
Breast only (*n* = 206)	168 (82%)	**42%**	38 **(58%)**	**0.0161**
Neither (*n* = 149)	135 (91%)	34%	14 (22%)	0.0553

**General ovarian cancer awareness**				
Yes (n = 154)	134 (87%)	34%	20 (31%)	0.6352
No (n = 306)	262 (86%)	66%	44 (68%)	
**General symptom awareness**				
Yes (n = 211)	181 (86%)	45%	30 (46%)	0.8807
No (n = 247)	213 (86%)	53%	34 (52%)	
**Matched symptom awareness**				
Yes (N = 183)	160 (87%)	40%	23 (35%)	0.4445
No (N = 275)	234 (85%)	59%	41 (63%)	
**Level of education**				
College/university/post-grad (n = 343)	304 (89%)	**76%**	39 **(60%)**	**0.0066**
<College (n = 122)	95 (78%)	24%	26 (40%)	
**First language**				
English (n = 359)	308 (86%)	77%	51 (78%)	0.8588
French (n = 81)	69 (85%)	17%	12 (18%)	
Other (n = 26)	23 (88%)	6%	3 (5%)	
**Self-reported ethnicity**				
Caucasian only (n = 342)	294 (86%)	74%	48 (74%)	1.000
French Canadian only (n = 57)	48 (84%)	12%	9 (14%)	0.6490
Multiple (n = 20)	17 (85%)	4%	3 (5%)	0.7077
**Household income**		(N=342)	(N=50)	
>$100,000 ($n$ = 168)	148 (88%)	43%	20 (40%)	0.6890
$75-99.9K ($n$ = 85)	74 (87%)	22%	11 (22%)	1.000
<$75K ($n$ = 139)	120 (86%)	35%	19 (38%)	0.6790
**Urban vs. rural**				
Urban (n = 402)	346 (86%)	87%	54 (83%)	0.3828
Rural (n = 59)	50 (85%)	13%	9 (14%)	0.8250

*respondents who did not consult a healthcare provider about the symptom/s they were experiencing; something else led to diagnosis

Table S7. Predictors of consulting a healthcare provider within three months of experiencing symptoms, among those who consulted.

Covariate		Univariable Analysis		Multivariable Analysis	
		OR (95%CI)*	*p*-value	OR (95%CI) &	*p*-value
**Age at diagnosis**					
	≤50 (*n* = 172)	Reference	---	Reference	---
	>50 (*n* = 226)	**2.03** (1.31,3.15)	**0.001**	**2.0** (1.27,3.15)	**0.003**
**Family history of OC**					
	Yes (*n* = 86)	Reference	---	---	---
	No (*n* = 301)	0.97 (0.57,1.65)	0.9		
**General OC awareness**					
	No (*n* = 261)	Reference	---	---	---
	Yes (*n* = 133)	1.15 (0.72,1.83)	0.56		
**General OC symptom awareness**					
	No (*n* = 212)	Reference	---	---	---
	Yes (*n* = 180)	0.88 (0.57,1.36)	0.55		
**Matched symptom awareness**					
	No (*n* = 233)	Reference	---	---	---
	Yes (*n* = 159)	0.91 (0.59,1.42)	0.69		
**Concerned about symptoms**					
	Not concerned (*n* = 117)	Reference	---	---	---
	Fairly concerned (*n* = 153)	0.81 (0.48,1.37)	0.43		
	Very concerned (*n* = 128)	1.18 (0.67,2.08)	0.57		
**Most concerning symptoms**					
	Menstrual irregularities (*n* = 37)	Reference	---	Reference	---
	Abdominal pain (*n* = 122)	**3.71** (1.71,8.05)	**<0.001**	**3.53** (1.61,7.73)	**0.001**
	Other/none (*n* = 160)	**2.26** (1.09,4.66)	**0.028**	1.94 (0.92,4.07)	0.08
	Persistent bloating (*n* = 78)	**3.52** (1.53,8.09)	**0.003**	**3.02** (1.29,7.05)	**0.011**
**Urban vs. rural**					
	Rural (*n* = 49)	Reference	---	---	---

Urban (n = 345)	0.59 (0.29,1.24)	0.16		
**Education**				
Up to/including high school (n = 48)	Reference	---	---	---
Certificate program (n = 47)	0.97 (0.39,2.39)	0.95		
College or above (n = 302)	0.89 (0.45,1.76)	0.74		
**Household income**				
<$75,000 ($n$ = 120)	Reference	---	---	---
$75,000-$99,999 (n = 74)	0.99 (0.53,1.85)	0.97		
>$100,000 ($n$ = 146)	1.14 (0.67,1.94)	0.62		
Prefer not to say (n = 58)	1.4 (0.69,2.87)	0.36		

OR=odds ratio; 95% CI=95% confidence interval (Tables S7-S10, S12). N=398; Time to consulting of <=3 Month=283; >3 months=115. OR for consulting a healthcare provider in ≤3 months (compared to >3 months) from *univariable and &multivariable logistic regression analysis. OC=ovarian cancer.

Table S8. Predictors of being diagnosed in ≤3 months (time to diagnosis as two categories).

Co-Variate		Univariable OR (95%CI; *p*-value)*	Multivariable OR (95%CI; *p*-value)[&]
**Age at diagnosis**			
	≤50	*Reference*	**NS
	>50	**1.90** (1.26-2.85; $p = 0.002$)	
**First healthcare provider**			
	Family doctor/other	*Reference*	NS
	ER/urgent care	**1.85** (1.1-3.13; $p = 0.021$)	
**Most concerning symptom**			
	Menstrual irregularities	*Reference*	*Reference*
	Abdominal pain	**3.87** (1.72-8.74; $p = 0.0011$)	**3.73** (1.55-8.98; $p = 0.0034$)
	Persistent bloating	**9.18** (3.71-22.72; $p < 0.001$)	**11.01** (4.08-29.67; $p < 0.001$)
	Other/none in particular	**3.65** (1.65-8.07; $p = 0.0014$)	**4.44** (1.86-10.6; $p < 0.001$)
**Whether first HCP ordered tests**			
	No tests and no referral	*Reference*	*Reference*
	No tests but referral made	0.55 (0.22-1.34; $p = 0.19$)	0.6 (0.23-1.6; $p = 0.31$)
	Tests ordered after further appts	0.88 (0.44-1.75; $p = 0.71$)	0.84 (0.4-1.77; $p = 0.65$)
	Tests ordered right away	**3.63** (2.02-6.52; $p < 0.001$)	**4.06** (2.14-7.72; $p < 0.001$)
**Province**			
	Ontario	*Reference*	*Reference*
	Alberta	0.91 (0.49-1.72; $p = 0.78$)	0.89 (0.43-1.83; $p = 0.75$)
	British Columbia	1.34 (0.71-2.52; $p = 0.36$)	1.29 (0.63-2.65; $p = 0.49$)
	Quebec	1.81 (0.99-3.33; $p = 0.055$)	**2.1** (1.06-4.18; $p = 0.034$)
	Saskatchewan	**0.38** (0.16-0.91; $p = 0.031$)	**0.38** (0.14,1; $p = 0.049$)
	Other	1.32 (0.59-2.94; $p = 0.5$)	1.78 (0.71-4.46; $p = 0.22$)
**Year of Diagnosis**			
	Up to 2010	*Reference*	*Reference*
	2011-2020	**1.95** (1.15-3.32; $p = 0.013$)	**1.97** (1.06-3.65; $p = 0.032$)
**Stage of Diagnosis**			
	I/II	*Reference*	NS

III/IV	**1.76** (1.16-2.68; *p* =0 .008)	
Unknown	3.84 (0.79-18.69; *p* = 0.096)	
**Ovarian cancer type at diagnosis**		
Non-epithelial cancer[1]	*Reference*	NS
High-grade serous	**3.47** (1.63-7.37; *p* = 0.0012)	
Non-high-grade serous epithelial cancer[2]	2.05 (0.91-4.6; *p* = 0.082)	
Other/unknown	**2.39** (1.07-5.31; *p* = 0.033)	

n=397 women with symptoms who sought medical help about their symptoms and time to diagnosis known (<=3 Month=238; >3 months=159). OR for time to diagnosis in ≤3 months (vs. >3 months) from *univariable and [&]multivariable logistic regression analysis. ** NS: not significant in multivariable analysis. [1] endometrioid, clear cell, low-grade serous. [2] germ cell, sex-cord stromal.

Table S9. Predictors of being diagnosed in <1 month and/or 1-3 months (time to diagnosis as three categories).

Covariate	Diagnosis in <1 Month		Diagnosis in 1-3 Months	
	**Univariable OR (95%CI; *p*-value)***	**Multivariable OR (95%CI; *p*-value)&**	**Univariable OR (95%CI; *p*-value)***	**Multivariable OR (95%CI; *p*-value)&**
**Age at diagnosis**				
≤50	*Reference*	NS	*Reference*	NS
>50	**1.78** (1.10-2.88; p=0.019)		**2.02** (1.24-3.32); p=0.005)	
**First healthcare provider (HCP)**				
Family doctor/other	*Reference*	*Reference*	*Reference*	*Reference*
ER/urgent care	#**2.58** (1.44-4.59; p=0.001)	#1.94 (0.98-3.85; p=0.056)	1.23 (0.65-2.34; p=0.526)	0.91 (0.44-1.89; p=0.800)
**Most concerning symptom**				
Menstrual irregularities	*Reference*	*Reference*	*Reference*	*Reference*
Abdominal pain	**2.83** (1.05-7.64; p=0.040)	1.79 (0.61-5.25; p=0.291)	**5.44** (1.75-16.9; p=0.003)	**5.87** (1.78-19.4; p=0.003)
Persistent bloating	**8.92** (3.09-25.7; p<0.001)	**7.30** (2.25-23.6; p<0.001)	**9.56** (2.82-32.4; p<0.001)	**12.3** (3.31-45.8; p<0.001)
Other/none in particular	**3.10** (1.19-8.12; p=0.021)	2.57 (0.89-7.39; p=0.081)	**4.46** (1.46-13.6; p=0.009)	**6.09** (1.85-19.9; p=0.003)
**Whether first HCP ordered tests**				
No tests and no referral	*Reference*	*Reference*	*Reference*	*Reference*
No tests but referral made	0.42 (0.12-1.45; p=0.169)	0.65 (0.17-2.47; p=0.531)	0.68 (0.23-2.05; p=0.497)	0.74 (0.23-2.41; p=0.618)
Tests ordered after further appts	0.79 (0.33-1.88; p=0.595)	0.95 (0.37-2.44; p=0.919)	0.97 (0.41-2.29; p=0.951)	0.91 (0.36-2.28; p=0.832)
Tests ordered right away	**3.66** (1.81-7.41; p<0.001)	**5.01** (2.28-11.0; p<0.001)	**3.59** (1.75-7.39; p<0.001)	**3.96** (1.80-8.69; p<0.001)
**Province**				
Ontario	*Reference*	*Reference*	*Reference*	*Reference*
Alberta	1.03 (0.47-2.23; p=0.940)	1.09 (0.46-2.58; p=0.836)	0.83 (0.39-1.74; p=0.619)	0.80 (0.35-1.83; p=0.591)

British Columbia	1.78 (0.86-3.68; p=0.122)	1.64 (0.71-3.79; p=0.250)	1.02 (0.48-2.15; p=0.968)	0.89 (0.38-2.06; p=0.783)
Quebec	##**2.88** (1.46-5.68; p=0.002)	##**3.21** (1.47-7.04; p=0.003)	1.02 (0.48-2.15; p=0.968)	1.14 (0.50-2.60; p=0.752)
Saskatchewan	0.29 (0.08-1.08; p=0.066)	0.33 (0.08-1.36; p=0.628)	0.45 (0.16-1.22; p=0.115)	0.48 (0.16-1.36; p=0.196)
Other	1.54 (0.59-3.95; p=0.371)	2.10 (0.69-6.44; p=0.194)	1.15 (0.46-2.93; p=0.763)	1.44 (0.51-4.12; p=0.494)
**Year of Diagnosis**				
Up to 2010	*Reference*	*Reference*	*Reference*	*Reference*
2011-2020	1.79 (0.95-3.37; p=0.071)	1.60 (0.74-3.49; p=0.235)	**2.15** (1.10-4.21; p=0.025)	**2.56** (1.16-5.66; p=0.019)
**Stage of Diagnosis**				
I/II	*Reference*	*Reference*	*Reference*	*Reference*
III/IV	###**2.47** (1.46-4.18; p<0.001)	**1.89** (1.03-3.50; p=0.041)	1.29 (0.79-2.11; p=0.309)	1.05 (0.59-1.86; p=0.867)
Unknown	4.83 (0.84-27.8; p=0.078)	**7.97** (1.01-62.6; p=0.048)	3.18 (0.56-18.1; p=0.192)	6.83 (0.87-53.9; p=0.068)
**Ovarian cancer type at diagnosis**				
Non-epithelial cancer[1]	*Reference*	NS	*Reference*	NS
High-grade serous	**4.04** (1.53-10.6; p=0.005)		**2.98** (1.18-7.53; p=0.021)	
Non-high-grade serous epithelial cancer[2]	1.74 (0.60-5.03; p=0.308)		2.32 (0.87-6.17; p=0.093)	
Other/unknown	**2.92** (1.05-8.07; p=0.039)		1.93 (0.72-5.21; p=0.192)	

n = 397 women with symptoms who sought medical help about their symptoms and time to diagnosis known (<1 Month=121;1-3 months=117; >3 months=159). *OR for diagnosis in <1 month or 1-3 months (vs >3 months) from *univariable and &multivariable multinomial regression analysis #Those with ER/urgent care as first healthcare provider were also more likely to be diagnosed in <1 month vs. 1-3 months (univariable OR 2.09, p=0.017; multivariable OR 2.14, p=0.024). ##Respondents from Quebec were also statistically more likely to be diagnosed in <1 month vs. 1-3 months (univariable OR 2.83, p=0.005; multivariable OR 2.81, p=0.009). ###Respondents diagnosed with stage III/IV disease were also more likely to be diagnosed in <1 month vs. 1-3 months (univariable OR 1.91, p=0.024). [1]endometrioid, clear cell, low-grade serous;[2]germ cell, sex-cord stromal.

Table S10. Predictors of being offered genetic testing after an ovarian cancer diagnosis.

Co-variate		Univariable OR (95%CI; p-value)*	Multivariable OR (95%CI; p-value)[&]
**Age at diagnosis**			
	≤50	*Reference*	NS
	>50	**1.71** (1.12-2.6; p=0.013)	
**Province**			
	Alberta	*Reference*	*Reference*
	Ontario	1.21 (0.64-2.28; p=0.56)	0.85 (0.37-1.94; p=0.70)
	British Columbia	**2.68** (1.11-6.49; p=0.029)	1.75 (0.57-5.34; p=0.33)
	Quebec	1.62 (0.77-3.42; p=0.21)	1.05 (0.41-2.73; p=0.91)
	Saskatchewan	**0.38** (0.15-0.96; p=0.041)	**0.25** (0.08-0.85; p=0.026)
	Other	1.26 (0.52-3.08; p=0.61)	1.05 (0.36-3.06; p=0.93)
**Year of Diagnosis**			
	Up to 2010	*Reference*	NS
	2011-2015	**1.92** (1.03-3.59; p=0.039)	
	2016-2020	**2.55** (1.49-4.36; p<0.001)	
**Stage of Diagnosis**			
	I/II	*Reference*	*Reference*
	III/IV	**4.03** (2.59-6.26; p<0.001)	**1.86** (1.05-3.29; p=0.033)
	Unknown	1.73 (0.44-6.74; p=0.43)	1.43 (0.32-6.39; p=0.64)
**Ovarian cancer type at diagnosis**			
	Non-high-grade serous epithelial cancer[2]	*Reference*	*Reference*
	High-grade serous	**8.23** (3.94-17.18; p<0.001)	**6.61** (2.96-14.75; p<0.001)
	Non-epithelial cancer[1]	**0.04** (0.01-0.13; p<0.001)	**0.04** (0.01-0.16; p<0.001)
	Other	**0.49** (0.25-0.96; p=0.038)	**0.45** (0.22-0.92; p=0.03)
**Family history of OC**			
	No	*Reference*	*Reference*
	Yes	**2.84** (1.49-5.39; p=0.0012)	**2.31** (1.08-4.93; p=0.031)
**First-degree relative with OC[#]**			

No	*Reference*	---
Yes	1.63 (0.55-4.84; p=0.38)	
**Family history of breast cancer**		
No	Reference	NS
Yes	**1.65** (1.08-2.53; p=0.021)	
**Family history of related cancers**[##]		
No	*Reference*	---
Yes	1.44 (0.94-2.2; p=0.094)	
**Travel time to surgery**		
Surgery took place <30 mins from home	*Reference*	---
Surgery took place between 30 mins to 1 hour from home	1.13 (0.65-1.98; p=0.66)	
Surgery took place >1 hour from home	0.81 (0.47-1.38; p=0.43)	

n = 500; all respondents, with the exception of those who had genetic testing performed prior to diagnosis, do not wish to be tested, can't remember or where timing of testing is not provided (Yes=383, No=117). OR for being offered genetic testing after diagnosis from *univariable and &multivariable logistic regression analysis. [1]endometrioid, clear cell, low-grade serous. [2]germ cell, sex-cord stromal. [#]e.g. mother, sister, daughter. [##]e.g. colorectal, uterine, pancreatic, prostate.

Table S11. Overview of treatments received & variations in use.

Co-variate	Surgery	Chemo	PARP inhibitors	Radiation	Anti-angiogenic	Aromatase inhibitor	Anti-estrogen	Immune therapy
**% of respondents**	**92%**	**86%**	**19%**	**11%**	**11%**	**9%**	**6%**	**4%**
**Age at diagnosis**								
≤50 (N=193)	94%	75%	16%	12%	8%	16%	8%	4%
>50 (N=332)*	90%	93%	20%	11%	13%	5%	4%	3%
p-value	---	**<0.0001**	0.2558	---	0.0797	**< 0.0001**	0.0522	---
**Stage of Diagnosis**								
I/II (N=200)	90%	73%	4%	14%	2%	7%	6%	2%
III/IV (N=313)	93%	96%	28%	10%	17%	11%	5%	5%
p-value	---	**<0.0001**	**<0.0001**	0.1674	**< 0.0001**	0.1311	---	0.0845
**OC type**								
HGSC (N=243)	94%	98%	32%	7%	13%	2%	4%	4%
Non-HGSC epithelial[2] (N=112)	90%	84%	3%	17%	11%	23%	6%	4%
Non-epithelial[1] (N=42)	93%	50%	0%	9%	2%	28%	12%	2%
p-value	---	**<0.0001** *(HGSC vs others)*	**<0.0001** *(HGSC vs others)*	**0.0087** *(non-HGSC ep vs others)*	**0.0371** *(non-epithelial vs others)*	**< 0.0001** *(HGSC vs others)*	0.0655 *(non-epithelial vs others)*	0.5214
Genetic test result								
Negative/not tested (N=377)	85%	78%	7%	12%	8%	9%	5%	2%
Inconclusive result (N=48)	90%	92%	27%	4%	21%	6%	4%	6%
Mutation in *BRCA1/2* (N=77)	96%	99%	65%	5%	10%	3%	6%	8%
	97%	88%	21%	21%	24%	18%	9%	6%

Mutation in other gene (N=34) p-value	---	---	**<0.0001** *(BRCA vs others)*	---	---	---	---	---
Travel time to surgery								
<30 mins (N=259)	*See below*	85%	20%	9%	9%	8%	8%	7%
30 mins to 1 hour (N=115)		92%	20%	13%	15%	11%	3%	1%
>1 hour from home (N=112)		85%	17%	12%	10%	12%	3%	0
p-value *(<30 mins vs others)*		---	---	0.1579	---	0.1406	**0.0175**	**0.0156**
Travel time to specific treatment	N=486	N=452	N=98	N=56	N=53	N=47	N=26	N=18
At home or <30 mins	53%	63%	94%	55%	64%	94%	88%	67%
30 mins-1 hour	24%	23%	1%	29%	26%	6%	8%	6%
>1 hour	23%	14%	5%	16%	9%	0	4%	28%
Urban vs. rural								
Urban (N=477)	88%	82%	18%	11%	10%	9%	6%	4%
Rural (N=75)	84%	83%	13%	7%	11%	8%	3%	0%
p-value	0.3314	0.8337	0.2879	0.2931	0.7899	0.7772	0.2937	0.0782

*respondents who did not indicate whether they had surgery were excluded.

Table S12. Predictors of being offered a clinical trial.

Co-variate	Univariable OR (95%CI; p-value)*	Multivariable OR (95%CI; p-value)[&]
**Age at diagnosis**		
≤50	*Reference*	---
>50	0.81 (0.53-1.24; p=0.14)	
**Province**		
Alberta	*Reference*	*Reference*
Ontario	1.79 (0.84-3.84; p=0.13)	1.64 (0.72-3.78; p=0.24)
British Columbia	1.44 (0.6-3.47; p=0.41)	1.4 (0.54-3.6; p=0.49)
Quebec	**2.91** (1.29-6.57; p=0.01)	2.58 (1.06-6.28; p=0.038)
Saskatchewan	0.35 (0.07-1.7; p=0.19)	0.38 (0.07-2; p=0.25)
Other	1.16 (0.41-3.27; p=0.78)	1.32 (0.42-4.15; p=0.63)
**Year of Diagnosis**		
Up to 2010	*Reference*	---
2011-2015	1.13 (0.58-2.2; p=0.71)	
2016-2020	0.82 (0.46-1.47; p=0.5)	
**Stage of Diagnosis**		
I/II	*Reference*	*Reference*
III/IV	**4.05** (2.38-6.87; p<0.001)	**3.3** (1.83-5.96; p<0.001)
Unknown	0.73 (0.09-6.01; p=0.77)	0.69 (0.08-6.08; p=0.74)
**Ovarian cancer type at diagnosis**		
Non-epithelial cancer[1]	*Reference*	*Reference*
High-grade serous	**3.14** (1.17-8.44; p=0.023)	1.19 (0.39-3.6; p=0.76)
Non-high-grade serous epithelial cancer[2]	1.89 (0.65-5.47; p=0.24)	1.09 (0.34-3.46; p=0.88)
Other/unknown	1.48 (0.52-4.25; p=0.46)	0.74 (0.2-2.73; p=0.65)
**Genetic test result**		
Tested negative or not tested	*Reference*	*Reference*
Inconclusive genetic testing result	1.53 (0.74-3.17; p=0.25)	1.24 (0.57-2.69; p=0.59)
Mutation in *BRCA1* or *BRCA2* gene	**2.27** (1.3-3.95; p=0.004)	1.61 (0.88-2.96; p=0.13)
Mutation in other gene	**2.61** (1.19-5.75; p=0.017)	**2.5** (1.05-5.96; p=0.038)

**Urban/rural**		
Rural	*Reference*	---
Urban	1.71 (0.83-3.52; p=0.14)	
**Travel time to surgery**		
Surgery took place <30 mins from home	*Reference*	NS
Surgery took place between 30 mins to 1 hour from home	0.97 (0.57-1.65; p=0.91)	
Surgery took place >1 hour from home	**0.55** (0.31-0.98; p=0.042)	

n = 451; all respondents, with the exception of those who don't remember if they were asked (Yes=119, No=332). OR for being offered clinical trial from *univariable and &multivariable logistic regression analysis. [1]endometrioid, clear cell, low-grade serous. [2]germ cell, sex-cord stromal.

Table S13. Summary of post-treatment follow-up care.

	All respondents not currently being treated (N=331)	≤1 year since last treatment (N=112)	1-5 years since last treatment (N=127)	>5 years since last treatment (N=84)	p-value (<5 vs. >5 yrs)
**Timing of follow-up appointments**					
Every 3 months	30%	54%	30%	1%	
Every 6 months	23%	14%	40%	6%	---
Yearly	8%	1%	6%	20%	
Other	20%	28%	17%	14%	
N/A – discharged	19%	4%	6%	58%	
**Assessments performed**					
Questions about symptoms	51%	76%	73%	40%	**<0.0001**
Blood test	49%	68%	63%	55%	0.1041
Physical exam	46%	57%	66%	49%	**0.0377**
Questions about side effects	18%	38%	20%	7%	**<0.0001**
Radiological assessment	14%	24%	20%	6%	**0.0010**
Questions about emotional/ psychosocial impact	11%	20%	13%	7%	**0.0391**
